# Phylogenetic analysis of promoter regions of human *Dolichol kinase* (DOLK) and orthologous genes using bioinformatics tools

**DOI:** 10.1515/biol-2022-0591

**Published:** 2023-05-23

**Authors:** Nadia Farooqi, Ataur Rahman, Yasir Ali, Kishwar Ali, Muhammad Ezaz Hasan Khan, David Aaron Jones, Mouadh Abdelkarim, Farman Ullah, Fazal Jalil

**Affiliations:** Department of Zoology, Women Campus, University of Swat, 19120, Swat, Pakistan; Department of Botany, Laboratory of Plant Ecology and Dendrochronology, University of Malakand, Khyber Pakhtunkhwa, Pakistan; Department of Biotechnology, Faculty of Chemical and Life Sciences, Abdul Wali Khan University Mardan, 23200, Mardan, Pakistan; College of General Education, University of Doha for Science and Technology, Al Tarafa, Jelaiah Street, Duhail North, PO Box 24449 Doha, Qatar; College of Health Sciences, University of Doha for Science and Technology, Al Tarafa, Jelaiah Street, Duhail North, PO Box 24449 Doha, Qatar; Centre for Biotechnology and Microbiology, University of Swat, 19120, Swat, Pakistan

**Keywords:** CDGs, CNS, DOLK, N-glycosylation

## Abstract

The Dolichol kinase (DOLK) gene encodes the polytopic DOLK protein associated with the endoplasmic reticulum (ER) N-glycosylation pathway catalyzing the final step in the biosynthesis of dolichol phosphate. Dolichol phosphate is an oligosaccharide carrier required for N-glycosylation of DOLK protein, with its deficiency leading to a severe hypo glycosylation phenotype in humans which can cause congenital disorders of glycosylation and death in early infancy. The aim of the present study is to identify the phylogenetic relationship between human and ortholog species based on their conserved sequences in DOLK gene. Sequence alignment of DOLK was carried out in this study and the evolutionarily conserved regulatory sequences were identified using bioinformatics. Promoter sequence of human DOLK was compared with orthologous sequences from different organisms. Conserved non-coding sequences (CNS) and motifs in promoter regions were found by analyzing upstream promoter sequences of *Homo sapiens* DOLK and its orthologous genes in other organisms. Conserved sequences were predicted in the promoter regions in CNS1 and CNS2. Conserved protein sequences were also identified by alignment of the orthologous sequences. Organisms with similar gene sequences are assumed to be closely related and the ER N-glycosylation pathway is conserved in them.

## 1 Introduction

Metabolic disorders including congenital disorders of glycosylation (CDG) result from abnormal protein and lipid glycosylation in the endoplasmic reticulum (ER) and Golgi apparatus. Glycosylation is a co- and post-translational addition of oligosaccharides (glycans) which are important in supporting protein folding, stability, and cell–cell adhesion [1,2]. Genetic defects in the N-glycosylation pathway can result in multisystem disease. CDGs are inborn metabolic errors, which form a rapidly growing group of 170 rare familial inherited diseases [3,4]. A defect in Dolichol Kinase (DOLK; E.C. 2.7.1.108) was discovered in 2007 by Kranz et al. [5].

CDG patients present with seizures, muscle hypotonia, ataxia, failure to thrive, ophthalmologic anomalies, endocrine and coagulation abnormalities, dysmorphism, psychomotor, and intellectual disability. A rare case of dilated cardiomyopathy (DCM) was reported in one of the two families with DOLK deficiency (DOLK-CDG, MIM 610768). The patients present with multisystem muscular hypotonia, ichthyosis, nystagmus, epilepsy, and pulmonary infection. These cases result in death at early stages in infants [5,6] with liver involvement and those with cognitive delay [6].

A gene (SEC59) coding for DOLK was first identified in temperature-sensitive yeast cells. The mutant yeast, deficient in this protein, ceased dividing and became enlarged at the restrictive temperature of 37°C, with inactive and incompletely glycosylated secretory proteins accumulating in the cell [5]. Human DOLK gene (ID: 22845, 9q34.11) was characterized at the molecular level by Fernandez et al., Shridas and Waechter [7,8]. This gene was found to be located on chromosome No. 9, with the open reading frame encoding for a protein consisting of 538 amino acids [9,10].

DOLK catalyzes the final step in the biosynthesis of Dolichol phosphate (Dol-P). Its deficiency in this pathway was reported to cause a severe hypo glycosylation phenotype in humans [11]. Three variants (CM1110851, CM111850, and CM1110849) in the gene have been associated with DCM [9]. Two siblings with neurological disorder were reported to have a homozygous missense mutation (p.M1?; c.2T > C) in DOLK [12]. Four patients homozygous for either c.295TrA (99Cys > Ser) or c.1322ArC (441Tyr > Ser) mutations, died in early infancy [5]. Autosomal recessive DOLK mutations were reported in 11 young patients (5–13 years) with DCM [13]. Two newborn female siblings with DCM and sever ichthyosis were found to have novel compound heterozygous mutations in DOLK: c.951C > A (p.Tyr317Ter) and c.1558A. G (p.Thr520Ala) which resulted in their death [14,15]. Using next generation sequencing, patient’s fibroblasts were identified as having homozygous c.1447C > A (p.Q483K) DOLK mutations [16]. These patients demonstrated severe effects upon enzymatic functions verifying *in silico* predictions as damaging (SIFT score 0.003) and probably damaging (Polyphen-2 score 0.975) [17,18]. A novel DOLK mutation was identified in a child with neonatal asphyxia, ichthyoid rash, and congenital heart disease [19].

To our knowledge, there are few bioinformatics studies available on the evolutionary analysis of DOLK gene in various species. We selected this gene for our study to understand its evolutionary history, gene expression, its contribution to the pathogenesis of different genetic disorders including DCM and to identify the cis-regulatory elements. Therefore, we investigated whether evolutionary conserved sequences are present in DOLK gene of ortholog species. The current study was conducted to analyze the human DOLK and its orthologous genes in order to study the phylogenetic relationship and to find conserved non-coding sequences (CNS) in the promoter region across various species. A comprehensive analysis of tissue specific expression was performed with the help of available expression data in Ensembl for the DOLK gene. The use of various bioinformatics tools will help us better understand the expression and regulation of the DOLK gene across various species. Consequently, by understanding the regulation and evolution of the DOLK gene, this study may help improve the diagnosis and treatment of diseases caused by defects in the DOLK gene or the ER N-glycosylation pathway.

## 2 Methods

### 2.1 DOLK gene and its expression analysis

**Figure 1:**
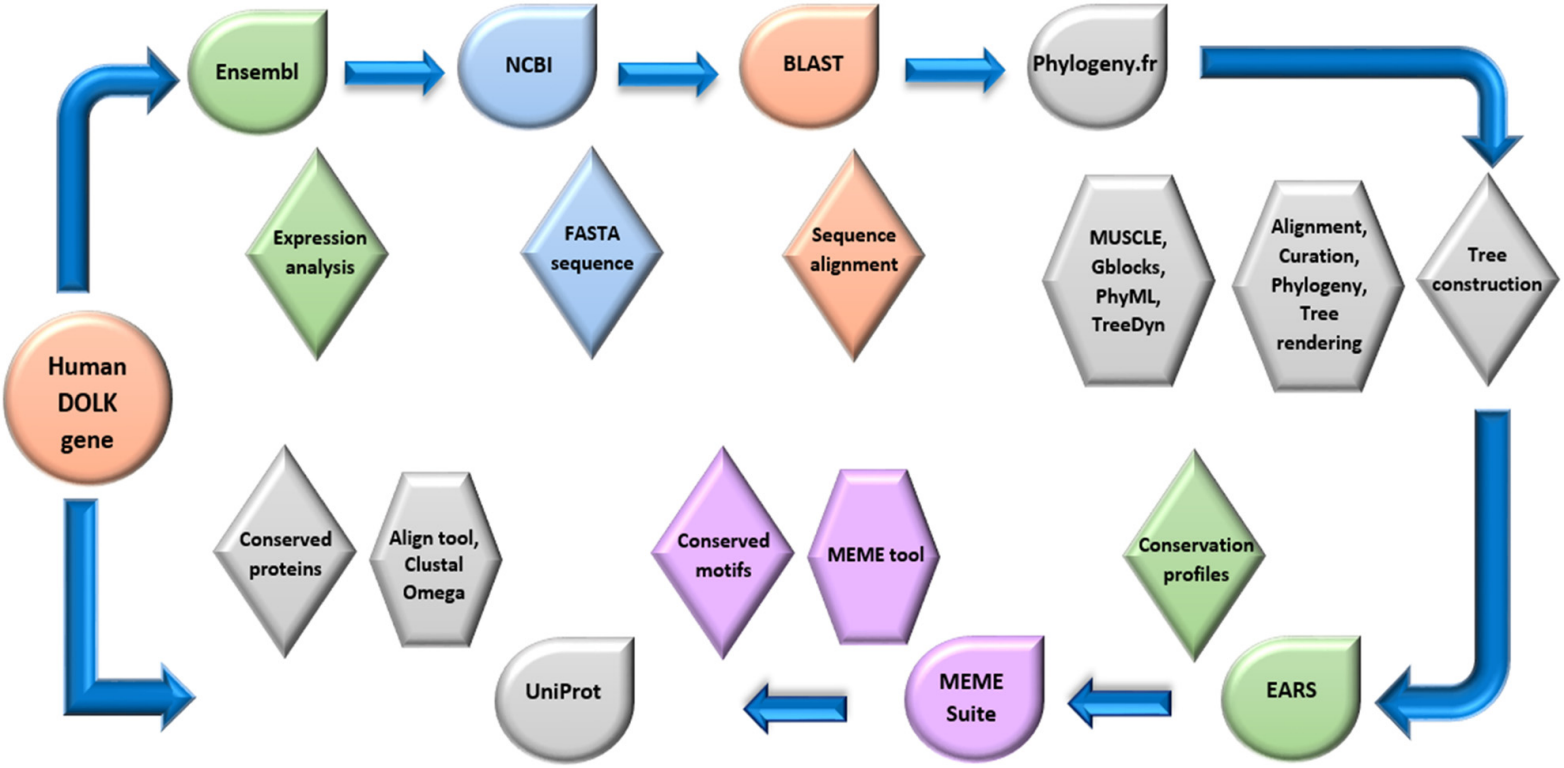
Workflow for identification of conserved sequences in the promoters of DOLK gene in orthologous species.

*In silico* expression profile of human DOLK gene was analyzed at the developmental stage with the help of the online database Ensembl (https://asia.ensembl.org/index.html). On the Ensembl homepage, our species of interest (Human) was chosen using the pull-down menu at the left of the search box and DOLK was typed in the search box. By clicking on the “Go” button, next page was opened. In the next step, DOLK (Human Gene) was selected. By clicking on gene expression on the right side, the expression profile of the DOLK gene was obtained. For identification of the DOLK gene ID, the National Center for Biotechnology Information (NCBI) database (https://www.ncbi.nlm.nih.gov/) was used. On the NCBI homepage, the pull-down menu was clicked to select the gene database. The gene name (DOLK) was typed in the text box and search button was clicked to obtain the gene ID. The workflow for the identification of conserved sequences in DOLK gene promoters of orthologous species is shown in Figure 1.

### 2.2 Screening for human DOLK gene and its orthologous gene sequences

The FASTA sequence of *Homo sapiens* DOLK gene was downloaded from NCBI database. The obtained sequence was then used as a query sequence in NCBI Basic Local Alignment Search Tool (BLAST) (http://blast.ncbi.nlm.nih.gov) to find the orthologous gene sequences, total score, query cover, sequence identity, and accession number for different species. On the NCBI BLAST homepage, Nucleotide BLAST was clicked, and query sequence (human DOLK sequence) was entered in the text area, using default parameters for analysis. After clicking the BLAST button, a table showing sequences of significant alignments was obtained (results are displayed in a new window). On clicking the download button, FASTA (complete sequence) was selected to obtain a file with FASTA sequences.

### 2.3 Sequence alignment and construction of the phylogenetic tree

Genes from the 15 closest orthologous members were selected based on their sequence identity. Using the accession number of *Homo sapiens* DOLK gene (query sequence) and its orthologous genes, the gene annotations and FASTA sequence were obtained from NCBI for the phylogenetic analysis of *Homo sapiens* and its orthologous species. The FASTA sequences of all the orthologous species were aligned using the bioinformatics tools (MUSCLE, Gblocks, PhyML, TreeDyn in Phylogeny.fr (http://www.phylogeny.fr) [20] and then the phylogenetic tree was constructed. Using the Phylogeny.fr server, the “One Click” Mode was selected and a set of sequences in FASTA format was pasted in the text box. By clicking the submit button, the sequence data were processed to construct the tree. The analysis was performed on the Phylogeny.fr platform and comprised four steps, namely, alignment, curation, phylogeny, and tree rendering.

First the sequences were aligned with MUSCLE using default settings. Second, the regions containing gaps and poorly aligned were removed with Gblocks using the default parameters. In the third step, the phylogenetic tree was reconstructed using the maximum likelihood method implemented in the PhyML program. In the fourth step, graphical representation and edition of the phylogenetic tree were performed with TreeDyn.

### 2.4 Searching CNS in the promoter regions

The comparative analysis was performed using FASTA sequences of 15 orthologous genes with the help of EARS software (http://nero.wsbc.warwick.ac.uk/tools/user_case_form.php) [21]. A template sequence (human DOLK sequence) was selected, to which all other orthologous sequences were compared. The orthologous sequences were used as comparison sequences. The species names were entered in each case. A cut off *P*-value of 0.1 and window size of 90 bp were selected. The resulting EARS file for each pair of species (human and one ortholog species) was analyzed individually and the location of significant peaks in the promoter region was detected for *Homo sapiens* DOLK gene and its orthologous sequence. Similarly, the cumulative EARS file was obtained for all the species.

The conserved motif sequences were identified in promoters using MEME Suite (https://meme-suite.org/meme/) [22]. This server performs motif-based sequence analysis and provides MEME tool. Using the FASTA sequences of DOLK gene and default parameters, three conserved motifs were identified in the promoters of the orthologous species, representing binding sites for the regulatory proteins. The results were obtained as MEME HTML output.

### 2.5 Aligning multiple sequences of DOLK protein

The DOLK protein sequences of different species were analyzed and aligned using the Clustal Omega algorithm. The UniProt database (https://www.uniprot.org/) [23] provides a multiple sequence alignment tool for proteins called “Align.” This tool runs the Clustal Omega program to find the similarity in the sequences being aligned. On the UniProt homepage, the Align tab in the toolbar was clicked to align the protein sequences with the Clustal Omega program. Protein sequences in FASTA format were entered in the text area. Output sequence order was selected similar to that of the input. The “Run Align” button was clicked to obtain the alignment results.

## 3 Results

### 3.1 Selection and expression analysis of DOLK gene in the species

**Figure 2:**
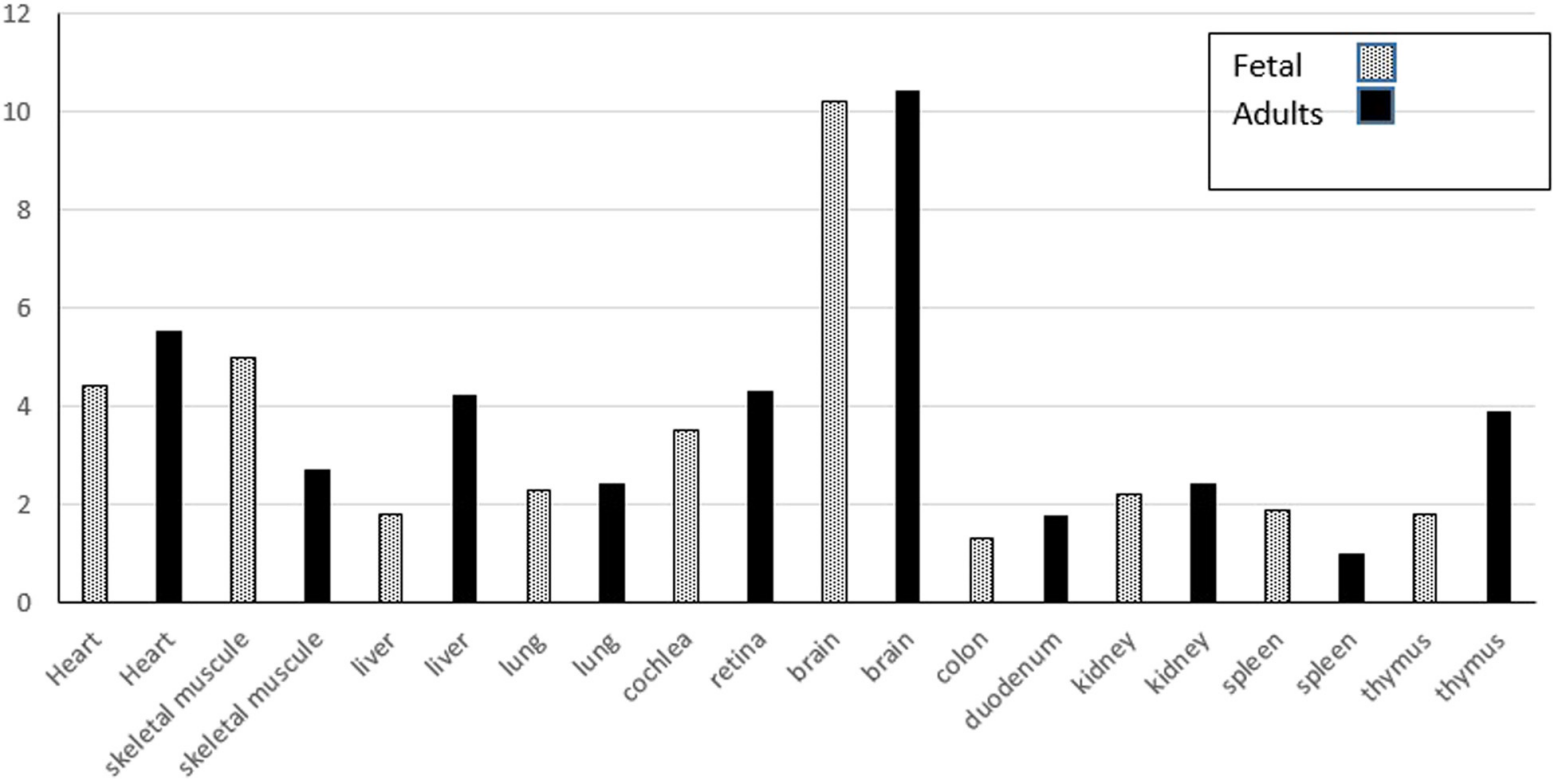
Human DOLK mRNA expression analysis in fetal and adult tissues shown as fold change in comparison to tissue with lowest expression level [9].

Gene expression analysis can help us to identify different genetic defects underlying human disorders. The Ensembl database was used to perform expression analysis of human DOLK gene in fetal and adult tissues. The highest expression level was found in fetal and adult brain tissue followed by adult heart, and fetal skeletal and heart tissues (Figure 2).

### 3.2 DOLK sequence in human and other ortholog species

The FASTA sequence of *Homo sapiens* DOLK gene downloaded from NCBI was used as query sequence in NCBI BLAST to obtain its orthologous gene sequences. Closely related organisms will have more similar sequences than distantly related organisms. Therefore, those species were selected which showed sequence identity >95% and were more closely related. The orthologous sequences were downloaded and saved in a word file. The total score, query cover, sequence identity, and accession number for different orthologous genes were obtained, as indicated in Table 1.

**Table 1:** Total score and other information for *DOLK* and orthologous genes in different species

S. no.	Source	Total score	Query cover (%)	Identity (%)	Accession
1.	*Homo sapiens*	4,071	100	100	NM_014908.3
2	*Gorilla gorilla*	4,015	99	99	XM_004048710.2
3	*Pan paniscus*	3,976	99	99	XM_008975907.1
4	*Nomascus leucogenys*	3,890	99	99	XM_003264225.3
5	*Pongo abelii*	3,888	99	99	XM_009244938.1
6	*Theropithecus gelada*	3,810	100	98	XM_025358577.1
7	*Piliocolobus tephrosceles*	3,808	99	98	XM_023216989.2
8	*Macaca mulatta*	3,494	91	98	NM_001266918.1
9	*Chlorocebus sabaeus*	3,792	99	98	XM_008005998.1
10	*Cercocebus atys*	3,783	99	98	XM_012038908.1
11	*Rhinopithecus bieti*	3,771	99	98	XM_017848286.1
12	*Colobus angolensis palliates*	3,762	99	98	XM_011931244.1
13	*Mandrillus leucophaeus*	3,736	98	98	XM_011971196.1
14	*Macaca fascicularis*	3,602	94	98	NM_001283633.1
15	*Aotus nancymaae*	3,533	99	96	XM_012458008.1

### 3.3 Sequence alignment and phylogenetic tree

**Figure 3:**
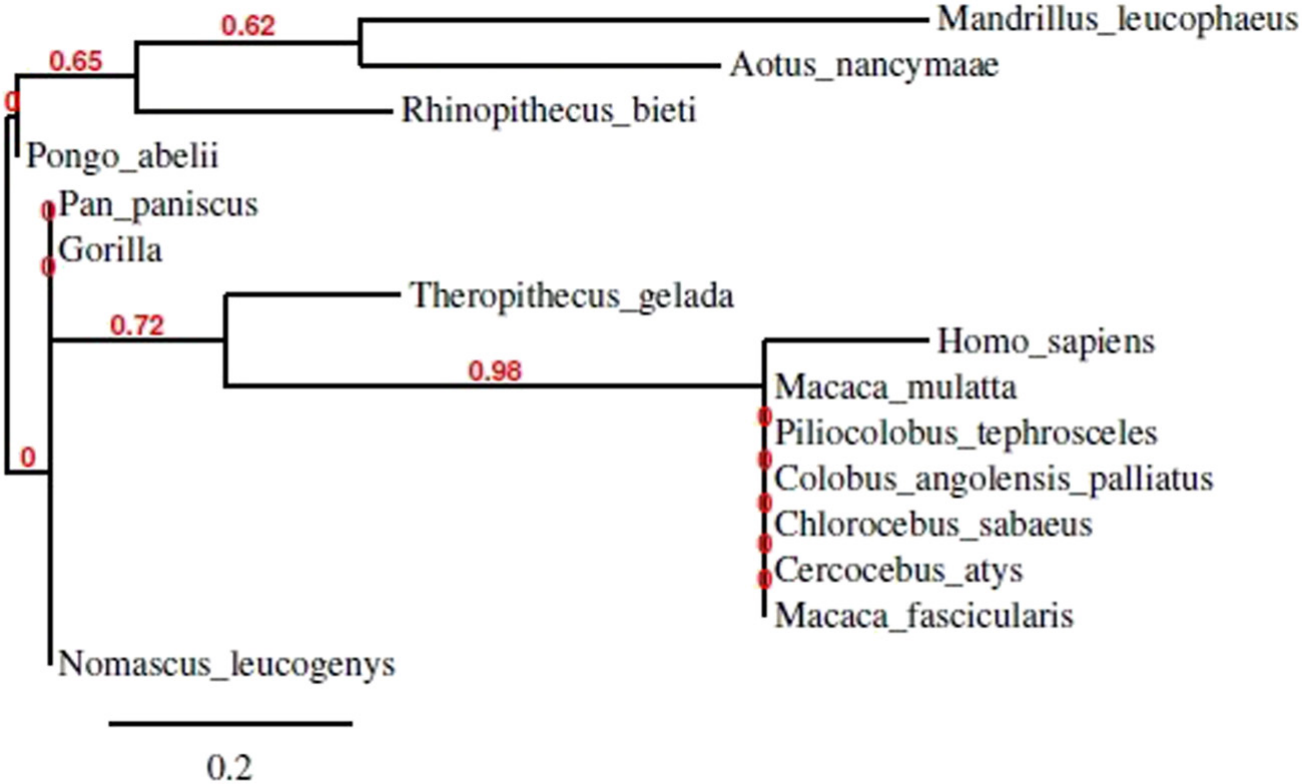
Phylogenetic tree for human DOLK gene and orthologous species using Phylogeny.fr.

The promoter regions of the DOLK orthologous genes showed close association across various species in order to conserve the biological role and expression of the gene. Phylogenetic analysis helped in the prediction of conserved promoter regions in the species. The phylogenetic tree was constructed by aligning FASTA sequences from human DOLK and 14 orthologous genes obtained from BLAST. It was constructed via the neighbor-joining method showing the ancestral relationship among species. The results of phylogenetic analysis are given in bootstrap values. The higher the bootstrap values, the more closely are the organism related to each other. The tree consisted of a clade with a higher bootstrap value (0.98) and a high confidence level, showing that human and other species are genetically and evolutionarily related to each other (Figure 3).

### 3.4 CNS in DOLK promoters

**Figure 4:**
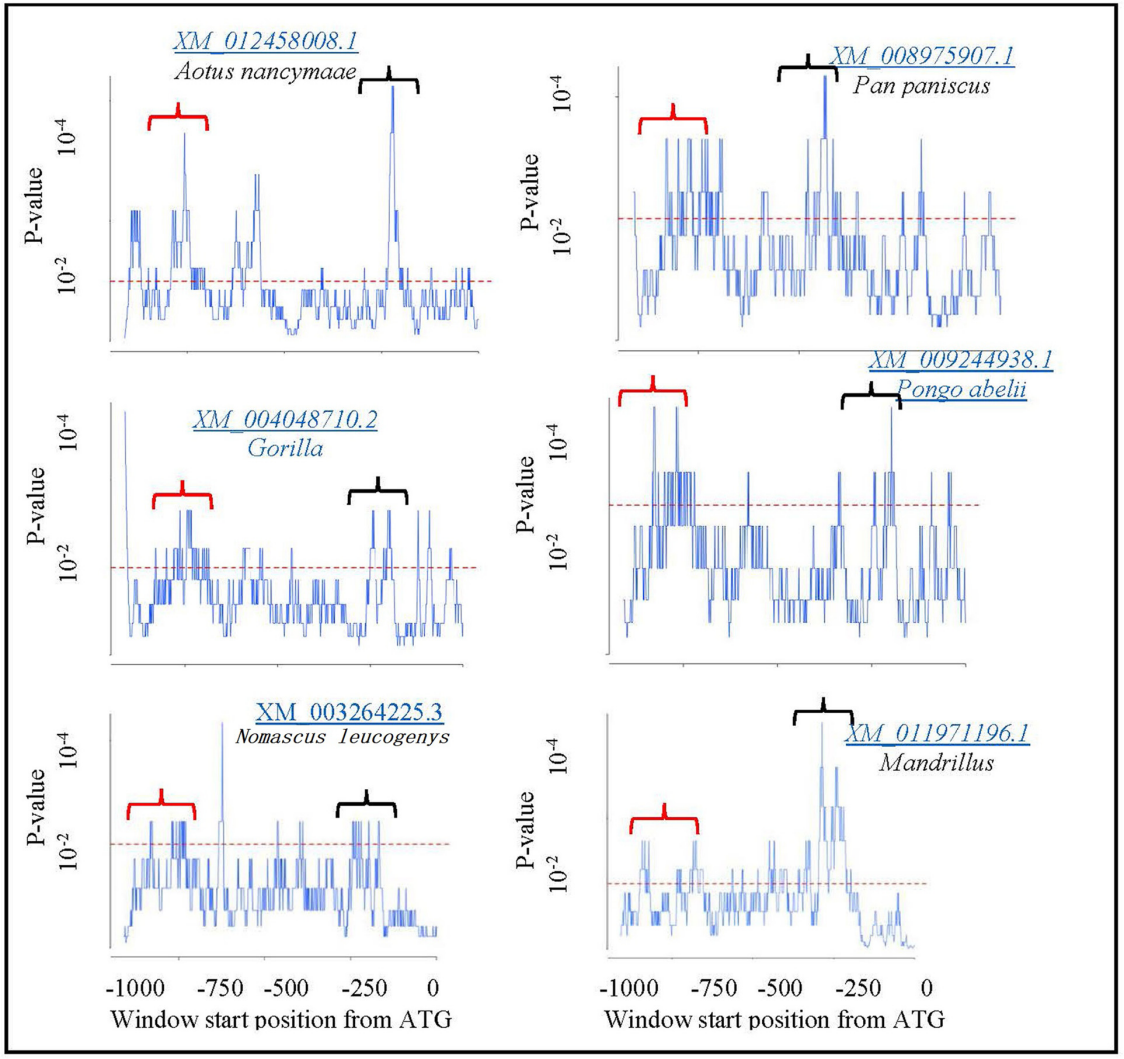
Identification of evolutionarily conserved sequences and individual conservation profile of human DOLK and orthologous promoters. The sequences upstream of the translation start codon were aligned using the EARS tool with a 90-base window length. Peaks above significance threshold of *P* = 0.1 (dotted red line) show a highly conserved match between species. Regions of conservativity highlighted with RED bracket = CNS1 and BLACK bracket = CNS2.

**Figure 5:**
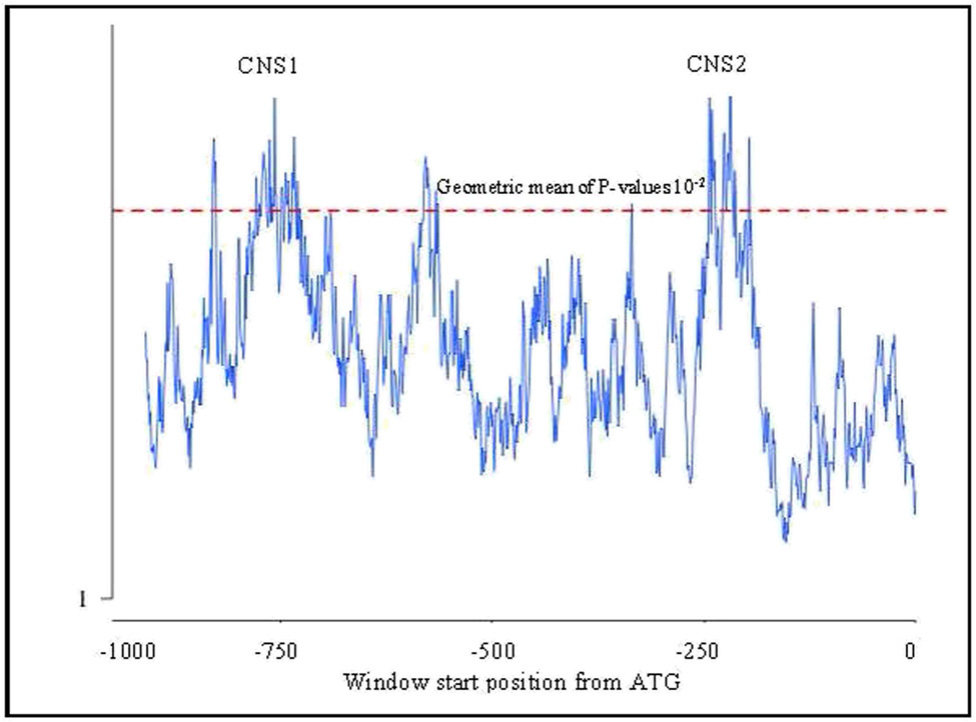
Cumulative conservation profile of human DOLK promoter and orthologous gene promoters from other species.

Promoter regions are functionally important for gene expression and are conserved during evolution. Promoter regions consisting of conserved sequences showed significant conservation between human and other species. EARS tool was used to compare the orthologous sequences to identify conserved regions in all the orthologs. After submitting sequences, EARS analysis was performed for each pair of species, and individual results (Figure 4) and cumulative results (Figure 5) were obtained. Cumulative results highlighted the conserved regions in all the species. In this study, two peaks were found above the significance threshold (*P* = 0.1) indicating a highly conserved match between human and other species. CNS1 and CNS2 were predicted in the promoter regions (Figure 5).

The promoter sequences upstream from the translation start codon were aligned using the EARS tool to identify the evolutionary conserved regulatory regions and a cumulative conservation profile was constructed for human DOLK promoter and promoters from all other orthologs (Figure 5).

Identification of the conserved motifs in the promoters was performed using MEME tool. A conserved motif in human *DOLK* promoter was found between positions −624 and −673. The second and third motifs were revealed in the promoter between positions −96 and −145, and between positions −560 and −609, respectively. Similarly, the motifs for other species were identified in their promoters. The sequences of these motifs in the orthologous species are shown as colored sequences in Supplementary File 1. These conserved motifs within the promoter of *DOLK* may represent the binding sites for the transcription factors, controlling the expression of *DOLK* gene. These results indicate that these promoter regions harboring conserved motifs are required for expression of *DOLK*.

### 3.5 Multiple protein sequence alignment

A set of FASTA sequences of the DOLK protein was pasted and aligned using the Clustal Omega program in UniProt database. The alignment results showed a significant similarity in the protein sequences of different species with only few different amino acid residues (Supplementary File 2). This shows that the DOLK protein is highly conserved in these species suggesting their role in phylogenetic analysis.

## 4 Discussion

In humans, the CNS in DOLK promoter involved in the ER N-glycosylation pathway can also be found in other organisms such as *Gorilla* sp., *Pan paniscus*, *Nomascus leucogenys*, *Pongo abelii*, *Mandrillus leucophaeus,* and *Aotus nancymaae* indicating a close evolutionary relationship between humans and other eukaryotes which can be correlated using the findings of Levy-Ontman et al. [24]. Sequence information is a transmutable phenomenon in living organisms, which is why conserved sequence shows the presence of a conserved gene. In the case of cross species conservation, this would indicate that a particular sequence has been unchanged by evolution as mutations in a highly conserved region results in non-viable life forms or a form quickly eliminated through natural selection.

Bioinformatics tools have been used to identify protein sequences involved in the ER N-glycosylation pathway of the red algae *Porphyridium* sp. These protein sequences were compared with ortholog protein sequences of other red algae, green algae, diatoms, mammals, and yeast which led to the identification of 24 encoded-genes homologous for known protein sequences in their ER pathways suggesting this pathway is conserved in animals, plants, yeasts, etc. [24]. Using a comparative genome-wide bioinformatics analysis, it was found that by analyzing upstream promoter sequences of glutamine synthetase cytosolic isozyme GS1;5 from *Arabidopsis thaliana* and its orthologous genes in various plant species, it was possible to identify CNS in promoter region [25]. Forty-two different enzymes in the N-linked glycan synthetic pathway or interactive pathways were found deficient in different types of CDG-N-linked disorders and multiple-pathway disorders [26,27]. DOLK variants have also been reported in patients showing a phenotype which overlaps CDG and dystroglycanopathy (DGpathy), with defective N-glycosylation and reduced O-mannosylation [28]. Neuromuscular disorders such as DGpathies have abnormal glycosylation of the glycoprotein dystroglycan [26]. Moreover, four genes DOLK (OMIM 610746), DPM1 (OMIM 603503), DPM2 (OMIM 603564), and DPM3 (OMIM 605951) encode for important enzymes involved in Dolichol metabolism [28] and mutations in them can cause some CDGs [29]. DGpathies have recently been associated with these four proteins and reported patients present with DCM, muscular dystrophy, and CDG diseases [29]. CDG-DOLK deficiency and cardiac pathology were reported in nine patients in three unrelated Israeli families with novel homozygous DOLK mutations. The cardiac symptoms ranged from discrete, mild DCM to overt heart failure with death [15,30]. Various studies have shown that DOLK is one of the candidate genes causing CDG, and mutations in this gene have also been associated with other genetic disorders such as DCM [13,26].

The present study is helpful in understanding the N-glycosylation pathway in different species. The conserved sequences have important biological functions. Their identification in human and lab organisms such as mice can be useful in predicting DOLK-related genetic anomalies. Besides its importance in the study of human diseases, phylogenetic analysis has a key role in identifying and characterizing newly discovered pathogens. As outlined above, the current information on evolutionary analysis of DOLK gene indicates that there are many gaps in our knowledge. The regulatory aspects of the N-linked glycan synthetic pathway and its interconnection within protein networks are still not well understood. Further studies on CDG patients will aid in finding some of the missing genes which cause the disease. Molecular and biochemical techniques will further help in our understanding of this diverse group of genetic disorders. Genome-wide association studies can be used to identify variations in conserved sequences associated with these diseases. Moreover, mechanism-based studies will help in monitoring the medication responses and in novel treatments in the future.

## 5 Conclusion

In conclusion, this study shows that human DOLK is closely related to orthologous genes in other species and has been found to express throughout the body. DOLK expression was found to be higher in both fetal and adult brain tissue with lower but still significant expression in adult heart, and fetal skeletal and heart muscle tissues. Evolutionary analysis of regulatory sequences using phylogenetic approach revealed highly conserved sequences and motifs in DOLK promoter region of human and orthologous species. In addition, sequence alignment of the orthologous proteins indicated conserved sequences with only few different amino acid residues. Currently, little is known about the evolutionary conserved sequences of DOLK gene in human and orthologous species. Going forward, it is recommended to screen the transcription factors binding sites for the DOLK gene promoter and to study other genes for evolutionary analysis. Gene expression studies on animal models will help us better understand the disease mechanisms and its treatment.

## 6 Web resources

Ensembl, https://asia.ensembl.org/index.html (17 June 2022).

NCBI, https://www.ncbi.nlm.nih.gov/(24 June 2022).

BLAST, http://blast.ncbi.nlm.nih.gov (3 July 2022).

Phylogeny.fr, http://www.phylogeny.fr (19 July 2022).

Evolutionary Analysis of Regulatory Sequences (EARS), http://nero.wsbc.warwick.ac.uk/tools/user_case_form.php (15 September 2022).

MEME Suite, https://meme-suite.org/meme/(2 February 2023).

UniProt, https://www.uniprot.org/(30 January 2023).

## Supplementary Material

Supplementary Figure 1

Supplementary Figure 2
