# Supplementary material for: Phylogenetic analysis of promoter regions of human Dolichol kinase (DOLK) and orthologous genes using bioinformatics tools
Source: Open Life Sci. 2023 May 23;18(1):20220591. doi: 10.1515/biol-2022-0591 (PMC10224619; doi:10.1515/biol-2022-0591)
Supplement: Supplementary Figure 1 [file biol-2022-0591-sm1.pdf]

First motif

Name	Strand	Start	*p*-value	Sites		
Gorilla	+	620	1.87e-24	TCGGGAG<br>ATC	GAGACCAGCCTGACCAACATGGAGAAACA<br>CTGTCTCTACTAAAAATACAA	AATTAGC<br>CAG
Homo	+	624	1.87e-24	TCGGGAG<br>ATC	GAGACCAGCCTGACCAACATGGAGAAACA<br>CTGTCTCTACTAAAAATACAA	AATTAGC<br>CAG
Mandrillus	+	844	4.19e-23	TCAAGAG<br>ATC	GAGACCATCCTGGCCAACAAGGTGAAACC<br>CCGTCTCTACTAAAAACACAA	AAATTAG<br>CTA
Aotus	-	189	2.35e-22	CGGGAGT<br>TGG	GAGACCAGCCTGACCAACATGGAGAAACC<br>CCATCTCTACTAAAAATACAA	AATTAGC<br>TGG
Nomascus	+	605	4.65e-22	TCGGGAG<br>TTC	GAGACCAGCCTGACCAACATGGAGAAACA<br>CCGTCTACTAAAAATACAAAA	TTAGCCA<br>GGC
Theropithecus	+	9	5.71e-22	ATGGGTT<br>G	GAGACCAGCCTGGCCAACATGGTGAAACC<br>CTGTCTCTATTAAAAATACAA	GAAATTA<br>GTT
Pongo	+	626	8.43e-22	TCGGGAG<br>TTG	GAGACCAGCCTGACCAACATGGAGAAACA<br>CCGTCTCTACTAAAAATATAA	AATTAGC<br>CAA
Rhinopithecus	-	187	5.09e-21	TCAGGAG<br>TTT	GAGACCAGCCTGACCAACATGGAGAAACC<br>CTGTCGCTACTAAAAATACAA	AATTAGC<br>GCG
M.	-	208	3.54e-20	AGGGACT<br>GAA	CTAGGCTTTAGGGACAAGAAGATGAAATA<br>TTACATACGAAAGCTACAATT	CTCACAA<br>AGT
Cercocebus	-	329	3.54e-20	AGGGACT<br>GAA	CTAGGCTTTAGGGACAAGAAGATGAAATA<br>TTACATACGAAAGCTACAATT	CTCACAA<br>AGT
Macaca	-	156	3.54e-20	AGGGACT<br>GAA	CTAGGCTTTAGGGACAAGAAGATGAAATA<br>TTACATACGAAAGCTACAATT	CTCACAA<br>AGT
Colobus	-	325	4.49e-20	AGGGACT<br>GAA	CTAGGCTTTAGGGACAAGGAGATGAAATA<br>TTATATACGAAAGCTACAATT	CTCACAA<br>AGT
Chlorocebus	-	329	4.49e-20	AGGGACT<br>GAA	CTAGGCTTTAGGGACAAGAAGATGAAATA<br>TTATATACGAAAGCTACAGTT	CTCACAA<br>AGT
Piliocolobus	-	326	6.54e-20	AGGGACT<br>GAA	CTAGGCTTTAGGGACAAGGAGATGAAATA<br>TCATATACGAAAGCTACAATT	CTCACAA<br>AGT

Second motif

Name	Strand	Start	*p*-value	Sites		
Gorilla	-	92	2.91e-28	GCTACTC<br>AGG	AGGCTGGGACAGGAGAATCGCTTGAACCTGG<br>GAGGTGGAGGTTGCGGTGA	GCCGAG<br>ATCG
Homo	-	96	2.91e-28	GCTACTC<br>AGG	AGGCTGGGACAGGAGAATCGCTTGAACCTGG<br>GAGGTGGAGGTTGCGGTGA	GCCGAG<br>ATCG
Pongo	+	723	7.07e-26	CCTACTA<br>GAG	AGGCTGAGGCAGGAGAATCACTTGAACCTGG<br>GAGGCGGAGGTTGAAGTGA	GCCGAG<br>ATCG
Nomascus	+	700	7.07e-26	ACTACTA<br>GAG	AGGCTGAGGCAGGAGAATCACTTGAACCTGG<br>GAGGCGGAGGTTGAAGTGA	GCCGAG<br>ATCG
Rhinopithecus	-	93	2.99e-25	GCTACCA<br>GGG	AGGCTGAGGCAGGAGAATCGCTTGAACCTGG<br>GAGGCAGAGGTTGCGGTGA	GCCGAG<br>ATCG
Theropithecus	+	573	2.09e-24	TCTACTG<br>GGG	AGGCTGAGGCAGGAGAATCGCTTGAACCCGG<br>GAGGCAGAGGTTGCAGTGA	GCCGAG<br>ATGG
Aotus	-	92	1.53e-22	TCTCCTC<br>GGG	AGGCTGAGGCAGGAGAATTGCTTGAACCTGG<br>GAGGCGGAGATTTTGGTGA	GCTGAG<br>ATTG
Mandrillus	+	52	2.15e-21	GCTACTT<br>GGG	AGGCTGAGACAGGAGAATGGCATGAACCCAG<br>GAGGTGGAGCTTGCAGTGA	GCCGAG<br>ATCG
Chlorocebus	+	83	1.81e-20	AGGAGGG<br>AGG	GTGGTGGGAAGACCGAAGACCTTGGAGCTGC<br>AATGTGGAAAACGGAGGGA	GGCGTG<br>GTGG
Colobus	+	79	3.15e-20	NNNAGGG<br>AGG	GTGGTGGGAAGACCGAAGACCTTCGAGCTGC<br>AATGTGGAAAACGGAGGGA	GGCGTG<br>GTGG
Piliocolobus	+	80	3.15e-20	AGGAGGA<br>TGG	GTGGTGGGAAGACCGAAGACCTTCGAGCTGC<br>AATGTGGAAAACGGAGGGA	GGCGTG<br>GTGG
Cercocebus	+	83	1.10e-19	AGGAGGG<br>AGG	GTGGTGGGAAGACCAAAGACCTTCGAGCTGC<br>AATGTGGAAAACGGAGGGA	GGCGTG<br>GTGG

Third motif

Name	Strand	Start	*p*-value	Sites		
Gorilla	+	556	1.05e-24	GCGTGGT<br>GGC	TCATGCCTGTAATCCCAGCACTTTGGGAG<br>GCCGAGGCAGGTGGATCACCT	GAGGTCG<br>GGA
Homo	+	560	1.05e-24	GCGTGGT<br>GGC	TCATGCCTGTAATCCCAGCACTTTGGGAG<br>GCCGAGGCAGGTGGATCACCT	GAGGTCG<br>GGA
Pongo	+	562	1.33e-21	GCGTGGT<br>GGC	TTATGCCTGTAATCCCAGCACTTTGGGAG<br>GCCGAGGCGGGCGGATCACCT	GAGGTCG<br>GGA
Rhinopithecus	-	251	5.25e-21	GGATGGT<br>GGC	TCACACCTGTAATCCCAGCACTTTGGGAG<br>GCCGAGGTGGGCGGATCACCT	GAGGTCA<br>GGA
M.	+	304	5.97e-21	CTCGCCG<br>ACT	GATTCAATGCAAGAAAAGAACGATTCGAG<br>GCGTGGAGAGGGCAGAAACCG	CAAGCGC<br>GGC
Colobus	+	421	5.97e-21	CTCGCTG<br>ACT	GATTCAATGCAAGAAAAGAACGATTCGAG<br>GCGTGGAGAGGGCAGAAACCG	CAAGTGC<br>GGC
Chlorocebus	+	425	5.97e-21	CTCGCCG<br>ACT	GATTCAATGCAAGAAAAGAACGATTCGAG<br>GCGTGGAGAGGGCAGAAACCG	CAAGCGC<br>CGC
Macaca	+	252	5.97e-21	CTCGCCG<br>ACT	GATTCAATGCAAGAAAAGAACGATTCGAG<br>GCGTGGAGAGGGCAGAAACCG	CAAGCGC<br>GGC
Piliocolobus	+	422	5.97e-21	CTCGCTG<br>ACT	GATTCAATGCAAGAAAAGAACGATTCGAG<br>GCGTGGAGAGGGCAGAAACCG	CAAGCGC<br>GGC
Aotus	-	254	2.77e-20	GCGTGGT<br>GGC	TCATGCCTATAAGCCCAGCACTTTGGGAG<br>GCTGAGGCGGGCGGATCACCT	GAGGTCG<br>GGA
Mandrillus	+	782	8.86e-20	ACGTGGT<br>AGC	TCACGCCTGTAATCCCAGCACTTTGGGAG<br>GCCGAGGTAGGCGGATCACGA	GGTCAAG<br>AGA
Nomascus	+	541	1.08e-19	GCACGGT<br>GGC	TCATGCCTGTAATCCCAGCACTTTGGGAG<br>GCCGACATGGGCGGATCACCC	GAGGTCG<br>GGA
Cercocebus	+	425	1.60e-19	CTCGCCG<br>ACT	GATTCAATGCAAGAAAAGAACGATTCGAG<br>GCGTGGAGACGGCAGAAACCG	CAAGCGC<br>GGC
Theropithecus	+	81	1.64e-14	ACGTGGT<br>AGC	GGGCGCCTGTAATCCCAGCTATTCGGGAG<br>ACTGAGGCAGGAGAATCCTTT	GAACCCA<br>GGA
Pan	-	198<br>9	6.01e-12	GTAACAG<br>CTA	GGCCATCAGCAATATCAGGAGGTAGAGAG<br>GCAGAAGGAGATTGTCTATCT	GTGTAGT<br>GTA
