# Supplementary material for: Phylogenetic analysis of promoter regions of human Dolichol kinase (DOLK) and orthologous genes using bioinformatics tools
Source: Open Life Sci. 2023 May 23;18(1):20220591. doi: 10.1515/biol-2022-0591 (PMC10224619; doi:10.1515/biol-2022-0591)
Supplement: Supplementary Figure 2 [file biol-2022-0591-sm2.pdf]

CLUSTAL Omega multiple sequence alignment

```
MTRECPSPAPGPGAPLSGSVLAEAAVVFAVVLSIHATVWDRYSWCAVALAVQAFYVQYKW 60 Homo
MTRECPSPAPGPGAPLSGSVLAEAAVVFAVVLSIHATVWDRYSWCAVALAVQAFYVQYKW 60 Gorilla
MTRECPSPAPGPGAPLSGSVLAEAAVVFAVVLSIHATVWDRYSWCAVALAVQAFYVQYKW 60 Pan
MTRECPSPAPGPGAPLSGSVLAEAAVVFAVVLSIHATVWDRYSWCAVALAVQAFYVQYKW 60 Nomascus
MTRECLSPAPGPGALLSGSVLAEAAVVFAVVLSIHATVWDRYSWCAVALAVQAFYVQYKW 60 Pongo
MTRECPSPAPGPGAPLSGSVLAEAAVVFAVVLSIHATVWDRYSWCAVALAVQAFYVQYKW 60 Theropithecus
MTRECPSPAPGPGAPLSGSVLAEAAVVFAVVLSIHATVWDRYSWCAVALAVQAFYVQYKW 60 Piliocolobus
MTRECPSPAPGPGAPLSGSVLAEAAVVFAVVLSIHATVWDRYSWCAVALAVQAFYVQYKW 60 Macaca
MTRECPSPAPGPGAPLSGSVLAEAAVVFAVVLSIHATVWDRYSWCAVALAVQAFYVQYKW 60 Chlorocebus
MTRECPSPAPGPGAPLSGSVLAEAAVVFAVVLSIHATVWDRYSWCAVALAVQAFYVQYKW 60 Cercocebus
MTRECPSPAPGPGAPLSASVLAEAAVVFAVVLSIHATVWDRYSWCAVALAVQAFYVQYKW 60 Rhinopithecus
MTRECPSPAPGPGAPLSGSVLAEAAVVFAVVLSIHATVWDRYSWCAVALAVQAFYVQYKW 60 Colobus
MTRECPSPAPGPGAPLSGSVLAEAAVVFAVVLSIHATVWDRYSWCAVALAVQAFYVQYKW 60 Mandrillus
MTRECPSPAPGPGAPLSGSVLAEAAVVFAVVLSIHATVWDRYSWCAVALAVQAFYVQYKW 60 M.
MTRECPSPAPGPGAPLSGSVLAETAVVFAVVLSIHAAVWDRYSWCAVALAVQAFYVQYKW 60 Aotus

**.*****:************:************************************

DRLLQQGSAVFQFRMSANSGLLPASMVMPLLGLVMKERCQTAGNPFFERFGIVVAATGMA 120 Homo
DRLLQQGSAVFQFRMSANSGLLPASMVMPLLGLVMKERCQTAGNPFFERFGIVVAATGMA 120 Gorilla
DRLLQQGSAVFQFRMSANSGLLPASMVMPLLGLVMKERCQTAGNPFFERFGIVVAATGMA 120 Pan
DRLLQQGSAVFQFRMSANSGLLPASMVMPLLGLVMKERCQTAGNPFFERFGIVVAATGMA 120 Nomascus
DRLLQQGSAVFQFRMSANSGLLPASMVMPLLGLVMKERCQTAGNPFFERFGIVVAATGMA 120 Pongo
DRLLQQGSAVFQFRMSANSGLLPASMVMPLLGLVMKERCQAAGNPFFERFGIVVAATGMA 120Theropithecus
DRLLQQGSAVFQFRMSANSGLLPASMVMPLLGLVMKERCQAAGNPFFERFGIVVAATGMA 120 Piliocolobus
DRLLQQGSAVFQFRMSANSGLLPASMVMPLLGLVMKERCQAAGNPFFERFGIVVAATGMA 120 Macaca
DRLLQQGSAVFQFRMSANSGLLPASMVMPLLGLVMKERCQAAGNPFFERFGIVVAATGMA 120 Chlorocebus
DRLLQQGSAVFQFRMSANSGLLPASMVMPLLGLVMKERCQAAGNPFFERFGIVVAATGMA 120 Cercocebus
DRLLQQGSAVFQFRMSANSGLLPASMVMPLLGLVMKERCQAAGNPFFERFGIVVAATGMA 120Rhinopithecus
DRLLQQGSAVFQFRMSANSGLLPASMVMPLFGLVMKERCQAAGNPFFERFGIVVAATGMA 120 Colobus
DRLLQQGSAVFQFRMSANSGLLPASMVMPLLGLVMKERCQAAGNPFFERFGIVVAATGMA 120 Mandrillus
DRLLQQGSAVFQFRMSANSGLLPASMVMPLLGLVMKERCQAAGNPFFERFGIVVAATGMA 120 M.
DRLLQQGSAVFQFRMSANSGLLPASMVMPLFGLVMKERCQAAGNPFFERFGIVVAATGMA 120 Aotus

******************************:*********:*******************

VALFSSVLALGITRPVPTNTCVILGLAGGVIIYIMKHSLSVGEVIEVLEVLLIFVYLNMI 180 Homo
VALFSSVLALGITRPVPTNTCVILGLAGGVIIYIMKHSLSVGEVIEVLEVLLIFVYLNMI 180 Gorilla
VALFSSVLALGITRPVPTNTCVILGLAGGVIIYIMKHSLSVGEVIEVLEVLLIFVYLNMI 180 Pan
VALFSSVLALGITRPVPTNTCIILGLAGGVIIYIMKHSLSVGEVIEVLEVLLIFVYLNMI 180 Nomascus
VALFSSVLALGITRPVPTNTCVILGLAGGVIIYIMKHSLSVGEVIEVLEVLLIFVYLNMI 180 Pongo
VALFSSVLALGITRPVPTNTCVILGLAGGVIIYIMKHSLSVGEVIEVLEVLLIFVYLNMI 180Theropithecus
VALFSSVLALGITRPVPTNTCVILGLAGGVIIYIMKHSLSVGEVIEVLEVLLIFVYLNMI 180 Piliocolobus
VALFSSVLALGITRPVPTNTCVILGLAGGVIIYIMKHSLSVGEVIEVLEVLLIFVYLNMI 180 Macaca
VALFSSVLALGITRPVPTNTCVILGLAGGVIIYIMKHSLSVGEVIEVLEVLLIFVYLNMI 180 Chlorocebus
VALFSSVLALGITRPVPTNTCVILGLAGGVIIYIMKHSLSVGEVIEVLEVLLIFVYLNMI 180 Cercocebus
VALFSSVLALGITRPVPTNTCVILGLAGGVIIYIMKHSLSVGEVIEVLEVLLIFVYLNMI 180Rhinopithecus
VALFSSVLALGITRPVPTNTCVILGLAGGVIIYIMKHSLSVGEVIEVLEVLLIFVYLNMI 180 Colobus
VALFSSVLALGITRPVPTNTCVILGLAGGVIIYIMKHSLSVGEVIEVLEVLLIFVYLNMI 180 Mandrillus
VALFSSVLALGITRPVPTNTCVILGLAGGVIIYIMKHSLSVGEVIEVLEVLLIFVYLNMI 180 M.
VALFSSVLALGITRPVPTNTCVILGLAGGVIIYIMKHSLSVGEVIEVLEVLLIFVYLNMI 180 Aotus

*********************:**************************************
```

```
LLYLLPRCFTPGEALLVLGGISFVLNQLIKRSLTLVESQGDPVDFFLLVVVVGMVLMGIF 240 Homo
LLYLLPRCFTPGEALLVLGGISFVLNQLIKRSLTLVESQGDPVDFFLLVVVVGMVLMGIF 240 Gorilla
LLYLLPRCFTPGEALLVLGGISFVLNQLIKRSLTLVESQGDPVDFFLLVVVVGMVLMGIF 240 Pan
LLYLLPRCFTPGEALLVLGGISFVLNQLIKRSLTLVESQGDPVDFFLLVVVVGMVLMGIF 240 Nomascus
LLYLLPRCFTPGEALLVLGGISFVLNQLIKRSLTLVESQGDPVDFFLLVVVVGMVLMGIF 240 Pongo
LLYLLPRCFTPGEALLVLGGISFVLNQLIKRSLTLVESQGDPVDFFLLVVVVGMVLMGIF 240Theropithecus
LLYLLPRCFTPGEALLVLGGISFVLNQLIKRSLTLVESQGDPVDFFLLVVVVGMVLMGIF 240 Piliocolobus
LLYLLPRCFTPGEALLVLGGISFVLNQLIKRSLTLVESQGDPVDFFLLVVVVGMVLMGIF 240 Macaca
LLYLLPRCFTPGEALLVLGGISFVLNQLIKRSLTLVESQGDPVDFFLLVVVVGMVLMGIF 240 Chlorocebus
LLYLLPRCFTPGEALLVLGGISFVLNQLIKRSLTLVESQGDPVDFFLLVVVVGMVLMGIF 240 Cercocebus
LLYLLPRCFTPGEALLVLGGISFVLNQLIKRSLTLVESQGDPVDFFLLVVVVGMVLMGIF 240Rhinopithecus
LLYLLPRCFTPGEALLVLGGISFVLNQLIKRSLTLVESQGDPVDFFLLVVVVGMVLMGIF 240 Colobus
LLYLLPRCFTPGEALLVLGGISFVLNQLIKRSLTLVESQGDPVDFFLLVVVVGMVLMGIF 240 Mandrillus
LLYLLPRCFTPGEALLVLGGISFVLNQLIKRSLTLVESQGDPVDFFLLVVVVGMVLMGIF 240 M.
LLYLLPRCFTPGEALLVLGGISFVLNQLIKRSLTLVESQGDPVDFFLLVVVVGMVLMGIF 240 Aotus

************************************************************

FSTLFVFMDSGTWASSIFFHLMTCVLSLGVVLPWLHRLIRRNPLLWLLQFLFQTDTRIYL 300 Homo
FSTLFVFMDSGTWASSIFFHLMTCVLSLGVVLPWLHRLIRRNPLLWLLQFLFQTDTRIYL 300 Gorilla
FSTLFVFMDSGTWASSIFFHLMTCVLSLGVVLPWLHRLIRRNPLLWLLQFLFQTDTRIYL 300 Pan
FSTLFVFMDSGTWASSIFFHLMTCVLSLGVVLPWLHRLIRRNPLLWLLQFLFQTDTRIYL 300 Nomascus
FSTLFVFMDSGTWASSIFFHLMTCVLSLGVVLPWLHRLIRRNPLLWLLQFLFQTDTRIYL 300 Pongo
FSTLFVFMDSGTWASSIFFHLMTCVLTLGVVLPWLHRLIRRNPLLWLLQFLFQTDTRIYL 300Theropithecus
FSTLFIFMDSGTWASSIFFHLMTCVLSLGVVLPWLHRLIRRNPLLWLLQFLFQTDTRIYL 300 Piliocolobus
FSTLFVFMDSGTWASSIFFHLMTCVLSLGVVLPWLHRLIRRNPLLWLLQFLFQTDTRIYL 300 Macaca
FSTLFVFMDSGTWASSIFFHLMTCVLSLGVVLPWLHRLIRRNPLLWLLQFLFQTDTRIYL 300 Chlorocebus
FSTLFVFMDSGTWASSIFFHLMTCVLSLGVVLPWLHRLIRRNPLLWLLQFLFQTDTRIYL 300 Cercocebus
FSTLFVFMDSGTWASSIFFHLMTCVLSLGVVLPWLHRLIRRNPLLWLLQFLFQTDTRIYL 300Rhinopithecus
FSTLFVFMDSGTWASSIFFHLMTCVLSLGVVLPWLHRLIRRNPLLWLLQFLFQTDTRIYL 300 Colobus
FSTLFVFMDSGTWASSIFFHLMTCVLSLGVVLPWLHRLIRRNPLLWLLQFLFQTDTRIYL 300 Mandrillus
FSTLFVFMDSGTWASSIFFHLMTCVLSLGVVLPWLHRLIRRNPLLWLLQFLFQTDTRIYL 300 M.
FSTLFVFMDSGTWASSIFFHLMTCVLGLGVVLPWLHRLIRRNPLLWLLQFLFQTDTRIYL 300 Aotus

**************************************:*******************

LAYWSLLATLACLVVLYQNAKRSSSESKKHQAPTIARKYFHLIVVATYIPGIIFDRPLLY 360 Homo
LAYWSLLATLACLVVLYQNAKRSSSESKKHQAPTIARKYFHLIVVATYIPGIIFDRPLLY 360 Gorilla
LAYWSLLATLACLVVLYQNAKRSSSESKKHQAPTIARKYFHLIVVATYIPGIIFDRPLLY 360 Pan
LAYWSLLATLACLVVLYQNAKRSSSESKKHQAPTIARKYFHLIVVATYIPGIIFDRPLLY 360 Nomascus
LAYWSLLATLACLVVLYQNAKRSSSESKKHQAPTIARKYFHLIVVATYIPGIIFDRPLLY 360 Pongo
LAYWSLLATLACLVVLYQNAKRSSSESKKHQAPTIARKYFHLIVVATYIPGIIFDRPLLY 360Theropithecus
LAYWSLLATLACLVVLYQNAKRSSSESKKHQAPTIARKYFHLIVVATYIPGIIFDRPLLY 360 Piliocolobus
LAYWSLLATLACLVVLYQNAKRSSSESKKHQAPTIARKYFHLIVVATYIPGIIFDRPLLY 360 Macaca
LAYWSLLATLACLVVLYQNAKRSSSESKKHQAPTIARKYFHLIVVATYIPGIIFDRPLLY 360 Chlorocebus
LAYWSLLATLACLVVLYQNAKRSSSESKKHQAPTIARKYFHLIVVATYIPGIIFDRPLLY 360 Cercocebus
LAYWSLLATLACLVVLYQNAKRSSSESKKHQAPTIARKYFHLIVVATYIPGIIFDRPLLY 360Rhinopithecus
LAYWSLLATLACLVVLYQNAKRSSSESKKHQAPTIARKYFHLIVVATYIPGIIFDRPLLY 360 Colobus
LAYWSLLATLACLVVLYQNAKRSSSESKKHQAPTIARKYFHLIVVATYIPGIIFDRPLLY 360 Mandrillus
LAYWSLLATLACLVVLYQNAKRSSSESKKHQAPTIARKYFHLIVVATYIPGIIFDRPLLY 360 M.
LAYWSLLATLACLVVLYQNAKRSSSESKKHQAPTIARKYFHLIVVATYIPGIIFDRPLLY 360 Aotus

************************************************************

VAATVCLAVFIFLEYVRYFRIKPLGHTLRSFLSLFLDERDSGPLILTHIYLLLGMSLPIW 420 Homo
VAATVCLAVFIFLEYVRYFRIKPLGHTLRSFLSLFLDERDSGPLILTHIYLLLGMSLPIW 420 Gorilla
VAATVCLAVFIFLEYVRYFRIKPLGHTLRSFLSLFLDERDSGPLILTHIYLLLGMSLPIW 420 Pan
```

```
VAATVCLAVFIFLEYVRYFRIKPLGHTLRSFLSLFLDERDSGPLILTHIYLLLGMSLPIW 420 Nomascus
VAATVCLAVFIFLEYVRYFRIKPLGHTLRSFLSLFLDERDSGPLILTHIYLLLGMSLPIW 420 Pongo
VAATVCLAVFIFLEYVRYFRIKPLGHTLRSLLSLFLDERDSGPLILTHIYLLLGMSLPIW 420Theropithecus
VAATVCLAVFIFLEYVRYFRIKPLGHTLRSLLSLFLDERDSGPLILTHIYLLLGMSLPIW 420 Piliocolobus
VAATVCLAVFIFLEYVRYFRIKPLGHTLRSLLSLFLDERDSGPLILTHIYLLLGMSLPIW 420 Macaca
VAATVCLAVFIFLEYVRYFRIKPLGHTLRSLLSLFLDERDSGPLILTHIYLLLGMSLPIW 420 Chlorocebus
VAATVCLAVFIFLEYVRYFRIKPLGHTLRSLLSLFLDERDSGPLILTHIYLLLGMSLPIW 420 Cercocebus
VAATVCLAVFIFLEYVRYFRIKPLGHTLRSLLSLFLDERDSGPLILTHIYLLLGMSLPIW 420Rhinopithecus
VAATVCLAVFIFLEYVRYFRIKPLGHTLRSLLSLFLDERDSGPLILTHIYLLLGMSLPIW 420 Colobus
VAATVCLAVFIFLEYVRYFRIKPLGHTLRSLLSLFLDERDSGPLILTHIYLLLGMSLPIW 420 Mandrillus
VAATVCLAVFIFLEYVRYFRIKPLGHTLRSLLSLFLDERDSGPLILTHIYLLLGMSLPIW 420 M.
VAATVCLAVFIFLEYVRYFRIKPLGHTLRSLLSLFLDERDSGPLILTHIYLLLGMSLPIW 420 Aotus

******************************:*****************************

LIPRPCTQKGSLGGARALVPYAGVLAVGVGDTVASIFGSTMGEIRWPGTKKTFEGTMTSI 480 Homo
LIPRPCTQKGSLGGARALVPYAGVLAVGVGDTVASIFGSTMGEIRWPGTKKTFEGTMTSI 480 Gorilla
LIPRPCTQKGSLGGARALVPYAGVLAVGVGDTVASIFGSTMGEIRWPGTKKTFEGTMTSI 480 Pan
LIPRPCTQKGSLGGARALVPYAGVLAVGVGDTVASIFGSTMGEIRWPGTKKTFEGTMTSI 480 Nomascus
LIPRPCTQKGSLGGARALVPYAGVLAVGVGDTVASIFGSTMGEIRWPGTKKTFEGTMTSI 480 Pongo
LIPRPCTQKGSLGGARALVPYAGVLAVGVGDTVASIFGSTMGEIRWPGTKKTFEGTMTSI 480Theropithecus
LIPRPCTQKGSLGGARALVPYAGVLAVGVGDTVASIFGSTMGEIRWPGTKKTFEGTMTSI 480 Piliocolobus
LIPRPCTQKGSLGGARALVPYAGVLAVGVGDTVASIFGSTMGEIRWPGTKKTFEGTMTSI 480 Macaca
LIPRPCTQKGSLGGARALVPYAGVLAVGVGDTVASIFGSTMGEIRWPGTKKTFEGTMTSI 480 Chlorocebus
LIPRPCTQKGSLGGARALVPYAGVLAVGVGDTVASIFGSTMGEIRWPGTKKTFEGTMTSI 480 Cercocebus
LIPRPCTQKGSLGGARALVPYAGVLAVGVGDTMASIFGSTMGEIRWPGTKKTFEGTMTSI 480Rhinopithecus
LIPRPCTQKGSLGGARALVPYAGVLAVGVGDTVASIFGSTMGEIRWPGTKKTFEGTMTSI 480 Colobus
LIPRPCTQKGSLGGARALVPYAGVLAVGVGDTVASIFGSTMGEIRWPGTKKTFEGTMTSI 480 Mandrillus
LIPRPCTQKGSLGGARALVPYAGVLAVGVGDTVASIFGSTMGEIRWPGTKKTFEGTMTSI 480 M.
LIPRPCTQKGSLGGARALVPYAGVLAVGVGDTVASIFGSTMGEIRWPGTKKTFEGTMTSI 480 Aotus

********************************:***************************

FAQIISVALILIFDSGVDLNYSYAWILGSISTVSLLEAYTTQIDNLLLPLYLLILLMA   538 Homo
FAQIISVALILIFDSGVDLNYSYAWILGSISTVSLLEAYTTQIDNLLLPLYLLILLMA   538 Gorilla
FAQIISVALILIFDSGVDLNYSYAWILGSISTVSLLEAYTTQIDNLLLPLYLLILLMA   538 Pan
FAQIISVAVILIFDSGVDLNYSYAWILGSISTVSLLEAYTTQIDNLLLPLYLLILLMA   538 Nomascus
FAQIISVALILIFDSGVDLNYSYTWILGSISTVSLLEAYTTQIDNLLLPLYLLILLMA   538 Pongo
FAQIISVALILIFDSGVDLNYSYAWILGSISTVSLLEAYTTQIDNLLLPLYLLILLMA   538Theropithecus
FAQIISVALILIFDSGVDVNYSYAWILGSISTVSLLEAYTTQIDNLLLPLYLLILLMA   538 Piliocolobus
FAQIISVALILIFDSGVDLNYSYAWILGSISTVSLLEAYTTQIDNLLLPLYLLILLMA   538 Macaca
FAQIISVALILIFDSGVDLNYSYAWILGSISTVSLLEAYTTQIDNLLLPLYLLILLMA   538 Chlorocebus
FAQIISVALILIFDSGVDLNYSYAWILGSISTVSLLEAYTTQIDNLLLPLYLLILLMA   538 Cercocebus
FAQIISVALILIFDSGVDLNYSYAWILGSISTVSLLEAYTTQIDNLLLPLYLLILLMA   538Rhinopithecus
FAQIISVALILIFDSGVDLNYSYAWILGSISTVSLLEAYTTQIDNLLLPLYLLILLMA   538 Colobus
FAQIISVALILIFDSGVDLNYSYAWILGSISTVSLLEAYTTQIDNLLLPLYLLILLMA   538 Mandrillus
FAQIISVALILIFDSGVDLNYSYAWILGSISTVSLLEAYTTQIDNLLLPLYLLILLMA   538 M.
FAQIISVALILIFDSGVDLNYSYAWILGSISTVSLLEAYTTQIDNLLLPLYLLILLMA   538 Aotus
                   ********:*********:****:**********************************
```
